# A Research and Development Agenda for the Control and Elimination of Human Helminthiases

**DOI:** 10.1371/journal.pntd.0001646

**Published:** 2012-04-24

**Authors:** Jürg Utzinger

**Affiliations:** 1 Department of Epidemiology and Public Health, Swiss Tropical and Public Health Institute, Basel, Switzerland; 2 University of Basel, Basel, Switzerland

In this issue of *PLoS Neglected Tropical Diseases*, the Disease Reference Group on Helminth Infections (DRG4) has put forward a collection of eight reviews that, taken together, outline a compelling research and development agenda for the control and elimination of helminth diseases of humans (http://www.ploscollections.org/helminths [1]–[8]). Emphasis is placed on six major helminth infections: (i) soil-transmitted helminthiasis; (ii) schistosomiasis; (iii) lymphatic filariasis; (iv) onchocerciasis; (v) food-borne trematodiasis; and (vi) cysticercosis/taeniasis. Selection of these helminthiases is justified on multiple grounds. Firstly, as shown in Table 1, more than half of the world's population is at risk of one or several of these helminthiases, and hundreds of millions of people are currently infected. Secondly, consequences of the mainly long-term chronic infection include suffering, stigmatisation, subtle and gross morbidity (e.g., anaemia, limb deformations and blindness), and premature death, hence causing an intolerable global burden [9]–[17]. These features, in turn, exacerbate poverty [18]–[20]. Thirdly, there is growing commitment at all levels—from local communities to politicians, philanthropic organisations, and civil society—to control and eventually eliminate/eradicate the major human helminthiases.

**Table 1:** Helminth Infections Emphasised by DRG4 for Development of a Research Agenda for Control and Elimination.

Helminth Infection	Causative Agent(s)	At-Risk Population (Millions)	No. of People Infected (Millions)	No. of People with Morbidity (Millions)	No. of Deaths per Year (Thousands)	Global Burden (Thousand DALYs)	Reference(s)
Soil-transmitted helminthiasis							
Ascariasis	*Ascaris lumbricoides*	5,416	807–1,221	350	3–60	1,817–10,500	[10], [11], [17]
Trichuriasis	*Trichuris trichiura*	5,307	604–795	220	3–10	1,006–6,400	[10], [11], [17]
Hookworm infection	*Ancylostoma duodenale* and *Necator americanus*	5,346	576–740	150	3–65	59–22,100	[10], [11], [17]
Strongyloidiasis	*Strongyloides stercoralis*	n.d.	30–100	n.d.	n.d.	n.d.	[10]
Lymphatic filariasis	*Wuchereria bancrofti*, *Brugia malayi*, and *B. timori*	>1,000	120	43	0	5,777	[11], [14]
Schistosomiasis^a^	*Schistosoma haematobium*, *S. japonicum*, and *S. mansoni*	779	207	120	15–280	1,702–4,500	[11], [12]
Food-borne trematodiasis							
Clonorchiasis	*Clonorchis sinensis*	601	15.3	1.1	5.6	275	[13], [16]
Paragonimiasis	*Paragonimus* spp.	292	23.2	5.3	0.2	197	[13], [16]
Fascioliasis	*Fasciola gigantica* and *F. hepatica*	91	2.6	0.3	0	35	[13], [16]
Opisthorchiasis	*Opisthorchis felineus* and *O. viverrini*	80	8.4	0.3	1.3	74	[13], [16]
Intestinal fluke infections	*Echinostoma* spp., *Fasciolopsis buski*, *Metagonimus* spp., and *Heterophyidae*	n.d.	6.7	0.9	0	84	[13], [16]
Onchocerciasis	*Onchocerca volvulus*	120	37	n.d.	0.05 (in the OCP area)	484	[9], [14]
Cysticercosis/taeniasis	*Taenia solium* and *T. saginata*	n.d.	n.d.	n.d.	n.d.	>2,000	[15]

a Listed are the three main schistosome species parasitising humans; of lesser importance are *S. guineenisis* and *S. intercalatum* (both restricted to West and Central Africa) and *S. mekongi* (restricted to Cambodia and Lao PDR).

DALY, disability-adjusted life year; n.d., not determined; OCP, Onchocerciasis Control Programme.

Figure 1 shows that the six helminthiases are at distinctively different stages of control and elimination with geographical idiosyncrasies for some of them [21]. Several helminthiases have been targeted for elimination (e.g., lymphatic filariasis and onchocerciasis in the Americas), and progress made thus far gives hope that this goal can indeed be achieved by 2015 or 2020 if certain conditions are met [14], [21]. For schistosomiasis and soil-transmitted helminthiasis, discussions are escalating to shift the focus from control to elimination [22], [23], and in Africa, the African Programme for Onchocerciasis Control (APOC) is also moving towards this goal [24]. The pivotal role of research, coupled with teaching and training of strong cadres of researchers from endemic settings as essential backbones and platforms to design, implement, and adapt the control and elimination agenda for major helminthiases as well as other tropical diseases, cannot be emphasised enough [25]–[27].

**Figure 1:**
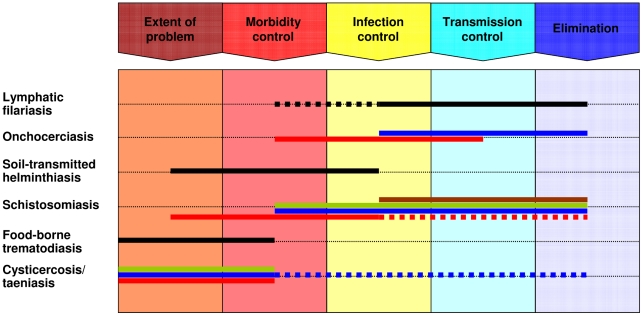
Stages of control/elimination of the six helminthiases emphasised by DRG4. The schematic representation shows the different stages of a control programme (from morbidity to transmission control), including the extreme ends (i.e., no control, extent of the problem yet to be determined on the far left; and elimination on the far right). Colour codes of horizontal lines: black, global; blue, Latin America and Caribbean; red, sub-Saharan Africa; brown, Middle East and North Africa; green, Asia. Dashed lines indicate that, in some counties within a sub-region (or globally), the control/elimination is less or more advanced than in the sub-region (or globally) overall.

In 2007/2008, the Special Programme for Research and Training in Tropical Diseases (TDR), based at and executed by the World Health Organization (WHO), and co-sponsored by the United Nations Children's Fund (UNICEF), the United Nations Development Programme (UNDP), the World Bank and WHO, initiated a network of disease-specific and thematic reference groups (DRGs/TRGs). DRG4 was particularly prolific, as witnessed in the collection of authoritative reviews presented here. This accompanying editorial summarises the goal, objectives and expected outcomes of the DRGs/TRGs global research “think tank”. Next, light is shed on the composition of DRG4, its mode of operation and its first outputs. It is then argued that *PLoS Neglected Tropical Diseases* is an appropriate outlet for the studies reviewed and synthesised by DRG4. Indeed, open access to the identified and ranked research priorities by all stakeholders is a major asset and is likely to reinforce the control and elimination agenda of the major helminthiases.

## A Global Research “Think Tank”

The idea to create a global network of DRGs and TRGs as a major international research “think tank” stems from the fourth external review of TDR that took place between February 2005 and May 2006 [28]. The role and operation of this “think tank” were incorporated into the 10-year strategic plan of TDR, which is endorsed by WHO. A key recommendation of the aforementioned external review was that TDR should shift its emphasis from pursuing a model of disease portfolio (“neglected diseases”) [29] to infectious diseases of poverty-related research needs (“needy populations”). As a result of this thinking, the 10-year strategy and underlying business plan of TDR recommended two strong pillars to position and further enhance the organisation's strategic advantages in a rapidly changing global landscape of funding, research and development: (i) knowledge management that should lead to the concept of stewardship, and (ii) capacity building that should foster empowerment [30].

Conceptualised in 2007/2008, a total of 10 reference groups, six of them with a disease-specific focus, and the remaining four with a thematic and cross-cutting emphasis, were initiated [2] (Table 2; see also Table S1 in [2]). Each reference group comprises 10–14 international experts. Groups are chaired and co-chaired by renowned scientists, at least one of whom is based in a developing country. A competitively selected young career research fellow is offered the opportunity to be part of the group, primarily pursuing research within the realm of the group, but also charged with some limited operational and managerial tasks to ascertain smooth operation of the groups. The reference groups are hosted by WHO country offices in Africa, Asia, and Latin America.

**Table 2:** Global Network of Disease-Specific Reference Groups (DRGs) and Thematic Reference Groups (TRGs).

DRG and TRG	Disease(s) or Thematic Focus	Hosting Institution(s) and Country(ies)
DRG1	Malaria	WHO Regional Office for Africa (AFRO), Republic of the Congo
DRG2	Tuberculosis, leprosy, and Buruli ulcer	WHO Country Office for the Philippines
DRG3	Chagas disease, human African trypanosomiasis, and leishmaniasis	WHO Country Offices for Sudan and Brazil
**DRG4**	**Helminthiases**	**African Programme for Onchocerciasis Control (APOC), Burkina Faso**
DRG5	Dengue and emerging viral diseases	WHO Country Office for Cuba
DRG6	Zoonosis and marginalised infectious diseases	WHO Regional Office for Easter and Mediterranean (EMRO), in collaboration with WHO Country Office for Egypt
TRG1	Social science and gender	WHO Country Office for Ghana
TRG2	Innovation and biotechnology platforms	WHO Country Office for Thailand
TRG3	Implementation and health systems research	WHO Country Office for Nigeria
TRG4	Environment, agriculture and human health	WHO Country Office for the People's Republic of China

In 2007/2008, the Special Programme for Research and Training in Tropical Diseases (TDR) set up a global network of DRGs and TRGs as an independent “think tank” of international experts. The groups met regularly to review, debate, and synthesise existing information, including stakeholder consultation. The ultimate aim was to establish and strengthen an evidence base on infectious diseases of poverty and cross-cutting themes, including identifying knowledge gaps and current research priorities.

As per TDR's latest 10-year strategy and vision, the global network of DRGs and TRGs operate within the guiding principles of knowledge management and capacity building, thus emphasising stewardship and empowerment. The specific objectives are to systematically review, debate, and synthesise available information and to define and rate research priorities. This is facilitated by regular meetings (it was initially planned that the groups would meet once a year for 3–5 days), usually in the hosting country of a specific group. Broad stakeholder consultation, usually at the outset of the meetings, was adopted as an integral part of the *modus operandi* of the groups.

## The Disease Reference Group on Helminth Infections (DRG4)

DRG4 initially comprised 14 individuals. The group is chaired by Sara Lustigman at the Lindsley F. Kimball Research Institute, New York Blood Center in New York, United States of America. Boakye A. Boatin serves as the co-chair, currently affiliated with two institutions, the Institute of Parasitology, McGill University (Montreal, Canada), and the Noguchi Memorial Institute of Medical Research, University of Ghana (Legon, Ghana). The career research fellow is based at the Council for Scientific and Industrial Research, Water Research Institute, in Ghana. The writing core team includes Sara Lustigman, Boakye A. Boatin, María-Gloria Basáñez (United Kingdom), and Roger K. Prichard (Canada). The geographical representation of the remaining nine members reveals two from Africa (Côte d'Ivoire and Ghana), two from Asia (Thailand and People's Republic of China), two from Latin America (Brazil and Peru), two from Oceania (Australia), and one from the Middle East (Egypt).

**Figure 2:**
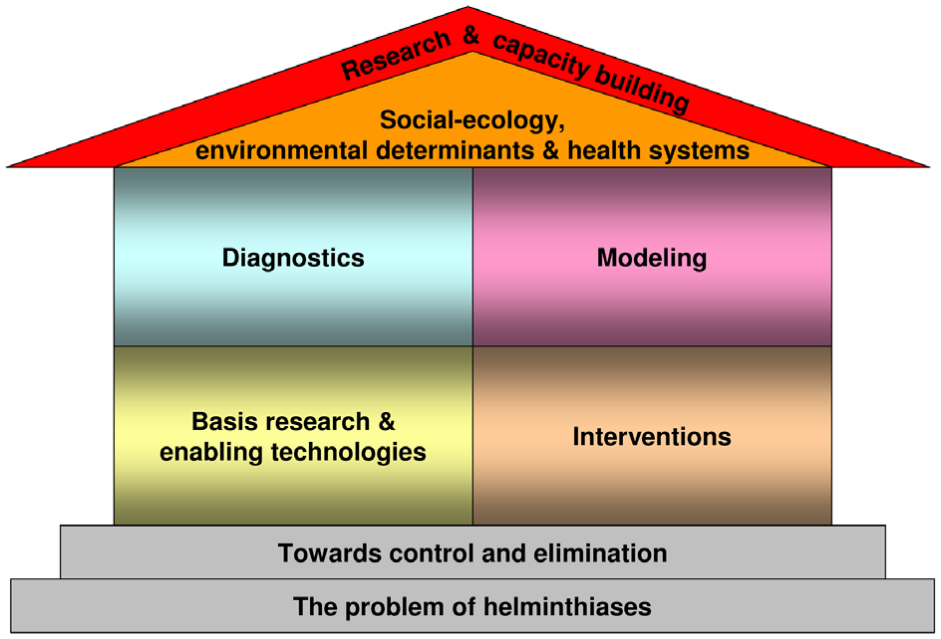
A research agenda for the control and elimination of major helminthiasis put forth by DRG4. This figure synthesises the key written outputs from DRG4, visualising this collection of eight reviews as a house (strong foundation, major building blocks and a two-layered roof).

At the outset, the group identified research gaps and pursued a multi-criteria assessment, including stakeholder consultation, and Delphi approach that ultimately resulted in a consolidated list of the 10 top research priorities. This forms the “white paper” of the DRG4, i.e., recognising and pursuing these priorities in order to achieve control and elimination of major human helminthiases. This “white paper” is co-authored by the entire group [2]. Next, an in-depth analysis outlines the problem of human helminthiases, emphasising the omnipresence of single and multiple species parasitic worm infections and their intricate relationship with poverty [1]. Visualising this collection as house, the reviews highlighting the problem of human helminthiases, and issues towards control and elimination, are like the foundation upon which the building is erected (Figure 2). Key proposed responses for the helminthiases control and elimination agenda are reviewed in four separate pieces that are the main building blocks of the house. These include basic research and enabling technologies [3], new and improved intervention tools and strategies, such as drugs, vaccines, and for some of the helminthiases also vector control [6], diagnostics [4], and mathematical modelling to inform policy and reinforce research [5]. Understanding social-ecological contexts, environmental determinants, and health systems forms the first layer of the roof [7]. Health research and capacity building in developing country settings is of such pivotal importance that it is layered on top of a well-built house, and hence reinforces the agenda [8].

It is, perhaps, interesting to note that key research and development (R&D) issues have already been discussed in connection with a malaria eradication research agenda (malERA). Facilitated by a 2-year broadly integrative and iterative process led by a core team, and assisted by various experts groups and involving more than 250 scientists, programme managers and decision-makers from the public and private sectors in a series of consultations, the malERA went live in a special issue of *PLoS Medicine* in early 2011 [27]. Similar R&D issues were identified in parallel by the malERA and the series presenting the research agenda for helminth infections of humans. Once the collection of malERA papers came out, they served as an inspiration to also publish the comprehensive R&D agenda pertaining to human helminthiases in a collection of reviews, and *PLoS Neglected Tropical Diseases* was deemed a suitable outlet. Both agendas feature basic science and enabling technologies [3], [31], diagnoses and diagnostics [4], [32], modelling [5], [33], and health systems issues [7], [34]. While the helminthiases agenda has a single chapter on interventions (due to the fact that, for instance, interventions such as anti-helminth vaccines are in earlier stages of development for use in humans [35]), in the malERA, there are three separate contributions for interventions: drugs, vaccines, and vector control [36]–[38]. This difference further emphasises the neglect in R&D funding for novel and repositioned intervention tools to control human helminth infections as opposed to malaria [39]. Additionally, the malERA views the issues of monitoring, evaluation, and surveillance (the latter further developed as “surveillance as an intervention”) of such great importance that space is given for a full contribution [40]. Together with a piece on cross-cutting issues for eradication, lessons learned from the first malaria eradication era in the 1950s and 1960s, and the aforementioned umbrella [27], the malERA certainly is a key resource for developing control and elimination/eradication agendas for human infectious diseases, including human helminthiases. However, the research agenda for helminthiases is unique in its contribution of a separate article fully considering the issues of capacity building for health research and disease control in endemic countries [8], which is absent in the malERA collection. Both collections therefore should be considered together as prime examples of major outputs produced by think tanks, driven by core writing teams and stakeholder engagement in the current global efforts to help control and eliminate poverty-related diseases [21].

## Going Live in *PLoS Neglected Tropical Diseases*

In terms of writing, the various DRGs and TRGs were charged to produce annual reports. Additionally, the groups were requested to have these reports further developed into stand-alone comprehensive Technical Report Series, to be published by the WHO. Unfortunately, at the time these reports were submitted to TDR, the organisation sailed through troubled waters, delaying internal processing and external peer review. Yet, there is new traction and it is hoped that the planned WHO Technical Report Series will soon come to bear. In the meantime, the Disease Reference Group on Zoonoses and Marginalized Infectious Diseases (DRG6) disseminated a most useful overview article through the open-access journal *Parasites & Vectors* [41].

The efforts made by DRG4 to produce no less than eight major reviews are commended and *PLoS Neglected Tropical Diseases* clearly is an appropriate outlet for this collection of articles. To wit, within 2–3 years after the launch of *PLoS Neglected Tropical Diseases* in late 2007, this open-access vehicle established itself as the leading peer-reviewed journal in tropical medicine. Two seminal papers published in 2005 and 2006 provided an initial list of 15 neglected tropical diseases, the majority of which were due to helminth infections [42], [43]. Indeed, all of the six helminthiases emphasised by DRG4 were part of the initial scope of *PLoS Neglected Tropical Diseases*. Meanwhile, the scope of the journal has broadened considerably [44], and now also includes a growing number of bacterial, protozoal, viral, and ectoparasitic infections, but helminthiases still figure prominently. Indeed, as shown in Table 3, more than 20% of the over 1,300 original articles, reviews, and front-matter pieces focus on helminth infections.

**Table 3:** Number and Percentage of Helminth-Related Articles Published in *PLoS Neglected Tropical Diseases*.

Search Strategy	No. (%) of Hits
*PLoS Neglected Tropical Diseases*	1335 (100)
Protozoan infections	294 (22.0)
**Helminth infections**	**277 (20.7)**
Viral infections	212 (15.9)
Bacterial infections	185 (13.9)
Fungal infections	11 (0.8)
Ectoparasitic infections	0
**Helminths (parasitic worms)**	**481 (36.0)**
**Trematodes**	**103 (7.7)**
**Nematodes**	**101 (7.6)**
**Cestodes**	**26 (1.9)**
Helminthic diseases	260 (19.5)
**Schistosomiasis**	**138 (10.3)**
**Lymphatic filariasis**	**57 (4.3)**
**Soil-transmitted helminthiasis**	**36 (2.7)**
**Onchocerciasis**	**34 (2.5)**
Echinococcosis	25 (1.9)
**Cysticercosis**	**23 (1.7)**
**Taeniasis**	**20 (1.5)**
Toxocariasis	7 (0.5)
Loiasis	6 (0.4)
**Food-borne trematodiasis**	**1 (0.1)**
Dracunculiasis	0
Important helminth species	
***Schistosoma mansoni***	**80 (6.0)**
**Hookworm**	**49 (3.7)**
***Schistosoma japonicum***	**39 (2.9)**
***Trichuris trichiura***	**32 (2.4)**
***Ascaris lumbricoides***	**31 (2.3)**
***Brugia malayi***	**21 (1.6)**
***Strongyloides stercoralis***	**17 (1.3)**
***Taenia solium***	**15 (1.1)**
***Wuchereria bancrofti***	**14 (1.0)**
***Onchocerca volvulus***	**13 (1.0)**
***Clonorchis sinensis***	**8 (0.6)**
***Fasciola hepatica***	**8 (0.6)**
***Opisthorchis viverrini***	**6 (0.4)**

Search performed on PubMed on March 15, 2012, using the advanced search builder. In a first step, the term “*PLoS Neglected Tropical Diseases*” was entered in the field “journal”, which revealed 1,335 hits. In subsequent steps, helminth-specific terms were added using the Boolean operator “AND”. Bold text in the table indicates parasites, parasitic infections, and diseases covered by the Disease Reference Group on Helminth Infections (DRG4).

## Lessons Learned and Next Steps

Sixty-five years after the landmark publication of Norman R. Stool entitled “This Wormy World” [45], it is clear that helminthiases are still widespread and continue to pose a huge public health problem. Indeed, a situation analysis reveals that more than half of the word's population is at risk of helminth infection, more than a billion people are currently infected, often with multiple species, and helminthiases are rife where poverty and malnutrition prevail, in the face of lack of access to basic infrastructure (e.g., clean water and sanitation) and hygiene [17], [20], [46]. These facts form the foundation of the current article collection [1], [2]. Responses on how to control and eventually eliminate human helminthiases require ethically, technically and scientifically sound research to improve current tools and strategies, as summarised in the present helminthiasis agenda [2]–[6]. Research and capacity building must accompany the entire process and innovation is key to developing and validating the next generation of tools and strategies [8].

For example, there is a need for developing rapid and inexpensive integrated mapping approaches for those helminthiases where the extent of the problem is not yet appreciated [47]. The recent development of high-resolution, spatially explicit global databases for helminthiases and other neglected tropical diseases provides an exciting new opportunity for targeting control interventions, and subsequent monitoring, evaluation, and surveillance [48], [49]. Importantly, once the emphasis shifts from morbidity control towards transmission control and finally local elimination, the need for highly accurate diagnostics tools must be stressed. Indeed, the diagnostics must be adapted to the current stage of a control programme [50]. Moreover, lessons learned from past successful helminthiases control and elimination programmes emphasise the need for integrated approaches with close collaboration between different sectors (e.g., health, education, and water) and long-term political commitment [51]–[53]. These issues must be seen in rapidly changing demographic, health systems, and social-ecological contexts [7]. A deep understanding at different spatial and temporal scales is mandatory so that some of the most ancient afflictions of humankind can be consigned to history in the not too distant future.
